# Targeting Inflammation to Prevent Cardiovascular Disease in Chronic Rheumatic Diseases: Myth or Reality?

**DOI:** 10.3389/fcvm.2018.00177

**Published:** 2018-12-11

**Authors:** Elena Bartoloni, Alessia Alunno, Valentina Valentini, Filippo Luccioli, Eleonora Valentini, Giuliana Maria Concetta La Paglia, Maria Comasia Leone, Giacomo Cafaro, Elisa Marcucci, Roberto Gerli

**Affiliations:** Rheumatology Unit, Department of Perugia, University of Perugia, Perugia, Italy

**Keywords:** rheumatic disease, inflammation, cardiovascular disease, atherosclerosis, biologic therapies, rheumatoid arthritis, psoriatic arthritis, ankylosing spondylitis

## Abstract

Evidence for increased risk of cardiovascular morbidity and mortality in chronic inflammatory rheumatic diseases has accumulated during the last years. Traditional cardiovascular risk factors contribute in part to the excess of cardiovascular risk in these patients and several mechanisms, including precocious acceleration of subclinical atherosclerotic damage, inflammation, and immune system deregulation factors, have been demonstrated to strictly interplay in the induction and progression of atherosclerosis. In this setting, chronic inflammation is a cornerstone of rheumatic disease pathogenesis and exerts also a pivotal role in all stages of atherosclerotic damage. The strict link between inflammation and atherosclerosis suggests that cardiovascular risk may be reduced by rheumatic disease activity control. There are data to suggest that biologic therapies, in particular TNFα antagonists, may improve surrogate markers of cardiovascular disease and reduce CV adverse outcome. Thus, abrogation of inflammation is considered an important outcome for achieving not only control of rheumatic disease, but also reduction of cardiovascular risk. However, the actual effect of anti-rheumatic therapies on atherosclerosis progression and CV outcome in these patients is rather uncertain due to great literature inconsistency. In this paper, we will summarize some of the main mechanisms linking the inflammatory pathogenic background underlying rheumatic diseases and the vascular damage observed in these patients, with a particular emphasis on the pathways targeted by currently available therapies. Moreover, we will analyze current evidence on the potential atheroprotective effects of these treatments on cardiovascular outcome pointing out still unresolved questions.

## Introduction

The long-term prognosis of chronic inflammatory rheumatic diseases (RDs), such as rheumatoid arthritis (RA), psoriatic arthritis (PsA), and ankylosing spondylitis (AS), is significantly influenced by increased risk of cardiovascular (CV) morbidity and mortality. In a large population-based, observational study, CV events resulted the third most frequent comorbidity in RA patients after depression and asthma (1). However, the evidence that screening and management of CV comorbidities in these patients is far from optimal deserves attention considering that high prevalence of atherosclerosis seems to occur yet in the earliest stages of the disease and also in young subjects free from CV risk factors, as demonstrated in particular in RA patients (2).

Chronic RDs and atherosclerotic endothelial damage share a similar inflammatory pathogenic background and multiple mechanisms contribute to subclinical atherosclerosis in these patients (3). It is demonstrated that disease-related inflammatory and immune mechanisms have a pivotal role in the pathogenesis of atherosclerosis and CV risk and that the contribution of traditional CV risk factors is at least as important as disease-specific factors (4). Indeed, prevalence of classic CV risk factors is higher in these patients in comparison to general population (5–8). In particular, hypertension, and diabetes mellitus represent two major factors to monitor in RD patients, both being associated with other CV comorbidities, disease activity and increased risk of CV events (5–9).

As inflammation is a cornerstone of the pathogenesis of systemic RDs and considering its pivotal role in driving all stages of atherosclerosis, it is compelling to hypothesize that controlling the pathways that induce synovial and systemic inflammation may provide benefit on CV risk in these patients (10). Although inconsistency in results between studies mainly due to different study design and different outcome measures, there are data suggesting that biologic therapies, in particular tumor necrosis factor-α inhibitors (TNFα-i), improve surrogate markers of subclinical atherosclerosis. Moreover, better control of RA activity has recently been associated with fewer CV events (11, 12). In a recent prospective study, failure in achieving disease activity control increased from 4- to 8-fold the risk to develop subclinical atherosclerosis and CV events at 1 year of follow-up (13). Although it is quite difficult to provide an actual long-term estimation of CV risk due to the lack of validated scores, tight, and sustained control of RD activity is necessary to effectively prevent CV disease development. Treat-to-target and abrogation of inflammation are now considered two main outcomes for achieving RD control. In addition, effective pharmacological treatment could favor physical activity, with consequent decrease of risk of obesity, diabetes, hypertension, and at least, CV disease. It is to note, however, that introduction of biologic agents is less frequent in RA patients with multiple concomitant comorbidities, although with active disease, and that some medications commonly used in these patients, such as corticosteroids (CS) and non-steroidal anti-inflammatory drugs, are known to enhance CV risk (14). In particular, some drugs may exert a dual effect. Indeed, if short-term CS treatment may lead to initial beneficial effect due to rapid suppression of inflammatory burden, it is well-known that long-term side effects of CS therapy may have a net adverse association with CV disease risk (15). Of consequence, the real effect of non-biologic and biologic therapy on CV risk and outcome in these patients is still uncertain.

In this perspective, a literature search was performed to identify articles investigating medium- and long-term effect of non-biologic and biologic therapies on subclinical atherosclerosis measures and CV outcome in patients with RA, PsA, and AS. Articles were identified in PubMed by using Mesh terms and keywords. Search was restricted to English language.

## Inflammation: A Link Between Atherosclerosis and Rheumatic Diseases

**Figure 1:**
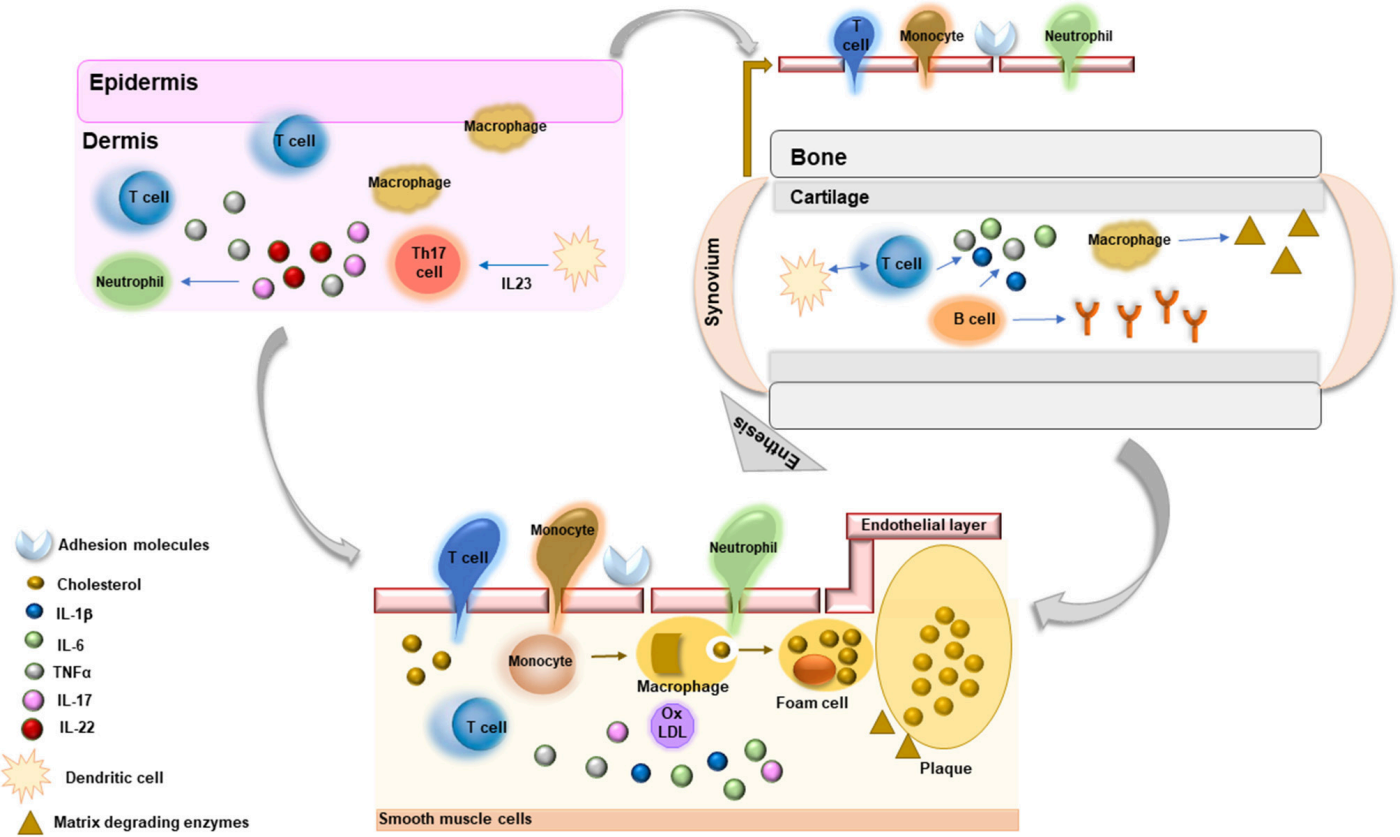
Common inflammatory mechanisms driving the pathogenesis of chronic rheumatic diseases and athrosclerosis.

The definite demonstration that atherosclerosis is a dynamic process greatly driven by inflammatory factors has highlighted interesting pathogenic links between atherosclerotic arterial wall damage and inflammatory mechanisms underlying the pathogenesis of systemic RDs (16) (Figure 1). Systemic inflammation contributes to all stages of atherosclerosis starting from activation of endothelial layer and recruitment of inflammatory cells within arterial layer to monocyte differentiation and foam cell formation, with subsequent plaque development. Moreover, these molecules promote apoptosis of arterial smooth muscle cells, matrix degradation, and fibrosis with subsequent destabilization and rupture of atherosclerotic plaques. Immune dysregulation, through the involvement of T lymphocytes, contributes to amplification of inflammatory response driving atherosclerotic damage. T helper (Th)1 cells, in particular, secrete several cytokines, such as interferon (IFN)γ, interleukin (IL)-2, IL-12, IL-18, and TNFα, which contribute to vascular endothelial damage and plaque progression (17). Interestingly, these cytokines, in particular TNFα, IL-6, and IL-18, have been associated with endothelial dysfunction, carotid atherosclerosis, CV morbidity, and risk of CV events and mortality in patients with systemic RDs (18). Among inflammatory biomarkers, C-reactive protein (CRP), IL-6, IL-1, and TNFα have been extensively studied and employed as predictive tools of CV risk and future CV events (16, 17, 19). Strong evidence supports the direct role of these molecules in contributing to atherogenesis by favoring endothelial dysfunction, vascular oxidative stress, foam cell formation, and atherosclerotic plaque destabilization (16, 17, 19). In addition, pro-inflammatory cytokines may induce atherosclerosis causing an alteration of lipid profile. In particular, TNFα and IL-6 have been shown to induce a pro-atherogenic profile and insulin resistance in patients with RDs (18).

Given the importance of pro-inflammatory cytokines in atherosclerosis and CV disease risk, effective modulation of inflammatory response in systemic RDs is expected to reduce risk and incidence of CV events and multiple pathways have been identified as potential therapeutic targets for the prevention and treatment of CV disease. In this setting, canakinumab, an inhibitor of IL-1β, was associated with significant reduction of recurrent CV events in patients with previous myocardial infarction and persistently elevated CRP levels (20). Three doses of canakinumab were tested and only the 150 mg dose reduced the relative risk of composite CV endpoint by 15%, mainly driven by a 24% reduction of relative risk of myocardial infarction. No significant reduction in CV death was observed and canakinumab was associated with an increased risk of fatal infection and sepsis. Surely, given the modest absolute clinical benefit, routine use of canakinumab in patients with previous myocardial infarction is not justified until more data are available.

On the other hand, in systemic RDs, randomized controlled trials (RCT)s of disease-modifying anti-rheumatic drugs (DMARDs), and biologic anti-cytokine therapies have not been powered to detect the impact of these agents on the modification of subclinical atherosclerosis and CV disease risk. Of consequence, data on the effect of these therapies on CV outcome in patients with RDs have been mainly driven by observational and pharmaco-epidemiological studies which suggest that close control of inflammation and disease activity in RDs may favorably affect some CV disease risk factors, reducing the rate of progression of subclinical atherosclerosis and the incidence of CV events (12) (Table 1).

**Table 1:** Effect of non-biologic and biologic drugs on CV risk in RD patients.

**Drug**	**Lipid profile**	**Metabolic syndrome**	**PWV**	**AIx**	**ED**	**IMT**	**Plaque**	**CV events**
**RHEUMATOID ARTHRITIS**								
HCQ	Improve							↓
MTX	Improve	↓						↓
TNFα-i	Worsen/neutral		↓		↓	↓		↓
TCZ	Worsen		↓	↓	↓	↔	↔	↔
ABT	Neutral		↔	↔		↔	↔	↓
RTX	Neutral		↔	↔		↓		↔
**PSORIATIC ARTHRITIS**								
TNFα-i	Worsen/neutral						
UST								↔
SEC								↔
**ANKYLOSING SPONDYLITIS**								
TNFα-i	Worsen/neutral			↔		↔	
SEC								↔

*HCQ, hydroxychloroquine; MTX, methotrexate; TNFα-I, tumor necrosis factorα inhibitors; TCZ, tocilizumab; ABT, abatacept; RTX, rituximab; UST, ustekinumab; SEC, secuckinumab; PWV, pulse wave velocity; AIx, augmentation index; ED, endothelial dysfunction; IMT, intima-media thickness; CV, cardiovascular*.

*↓, significantly decreased; ↔, no significant effect*.

## Do Anti-Rheumatic Therapies Lower the Risk of Accelerated Atherosclerosis?

Endothelial dysfunction, a potentially reversible step in atherosclerosis development, and structural vascular wall damage, assessed either as intima-media thickness (IMT) and carotid plaque, are considered important predictors of subsequent CV events in the general population as well as in patients with RA (21, 22). Multiple mechanisms, including systemic inflammatory burden, have been implicated in the pathophysiology of micro and macro-vascular endothelial dysfunction in patients with RDs and different methods are employed to detect precocious atherosclerosis in these patients (23). Thus, therapies reducing inflammation and disease activity are expected to improve vascular function and, possibly, arterial wall organic damage. In this setting, however, no definite conclusions can be driven on the effect of anti-rheumatic therapies on vascular endothelial function in inflammatory RDs. Multiple variables, including differences in study design, population enrolled, disease duration, instrumental technique employed in the assessment of subclinical atherosclerosis, length of follow-up, class of biologic drug used and concomitant therapies, hamper data interpretation and explain the high variability of study results. However, analysis of data derived by meta-analysis and systematic reviews, observational studies, and few RCTs allows to highlight some observations.

### Conventional DMARDs

- Hydroxychloroquine (HCQ) has been associated with lower risk of diabetes mellitus (24), a better lipid profile characterized by reduced low-density lipoprotein and trygliceride levels (25) and antithrombotic effect on platelet aggregation. Moreover, *in vitro* studies demonstrated a potential vasoprotective effect by reduction of vascular endothelial adhesion molecule expression (26). Despite this beneficial evidence on lipid and glucose homeostasis, no studies explored the effects of HCQ on surrogate markers of atherosclerosis. Interestingly, a recent meta-analysis demonstrated that patients with RA and systemic connective diseases assuming HCQ are characterized by a significant reduction of CV events in comparison to non-HCQ users (27).
- Methotrexate (MTX) has several favorable effects on markers of CV damage. In particular, MTX therapy has been associated with improvement in reverse cholesterol transport (28), reduction of foam cell formation (29), down-regulation of adhesion molecule expression on endothelial surface (30), and reduced risk of metabolic syndrome (31). Moreover, response to MTX therapy is associated with reduction of circulating cytokines, including TNFα, IL-6, and IL-1, which exert atherogenic activity. Effects of MTX on measures of subclinical atherosclerosis has been explored in few studies showing a favorable response in atherosclerosis progression (32–34). In a recent observational study, 6-month MTX monotherapy was associated with a more pronounced favorable effect on endothelial function in comparison to TNFα-i ± MTX in a cohort of RD patients (35). The effect was independent of disease activity improvement. However, the small number of patients enrolled and the method used to detect atherosclerosis progression (change in Reactive Hyperemic Index) suggest caution in data interpretation.

### TNFα Inhibitors

- Short and medium-term studies demonstrated that TNFα-i are effective in improving arterial stiffness, evaluated as reduction of pulse wave velocity (PWV), and endothelial dysfunction, expressed as improvement in flow-mediated vasodilation (FMD), in RA patients, thus suggesting a link between chronic inflammation and endothelial dysfunction and arterial stiffness (11, 36, 37).
- TNFα-i therapy is associated with prevention or reversion of IMT progression in RD patients responding to treatment in studies with up to 5-year follow-up (36). The effect on IMT seems more relevant in RA patients with early disease (38).
- A beneficial effect on measures of microvascular endothelial dysfunction has been depicted in a small cohort of AS patients following 1 month of etanercept therapy, thus suggesting that suppression of inflammation is associated with rapid reversal of microvascular dysfunction in these patients (39). On the other hand, no effect of TNFα-i treatment has been detected on arterial stiffness and augmentation index (AIx) in wider cohorts of AS patients, suggesting that different disease-specific mechanims may contribute to endothelial impairment (40).
- AIx, a composite measure of arterial stiffness and speed of reflected wave from peripheral vascular resistances, usually does not change following TNFα-i therapy (36, 37). Intriguingly, this may suggest that arterial stiffness, a surrogate measure of macrovacular function, is more sensitive to inflammatory burden in RDs in comparison to other vascular functional parameters.
- Different TNFα-i may exert different effects on subclinical atherosclerosis. In this setting, adalimumab and etanercept have been associated with significant reduction of arterial stiffness in RA patients, while no change in the same measure was detected following infliximab administration (40). However, the limited number of studies does not allow to demonstrate a clear class-specific effect of TNFα-i on endothelial function in these patients (11).

### Other Non-TNFα-i Therapies

Very few data are available on the effect of other non-TNFα-i targeted therapies on subclinical vascular endothelial damage. Inhibition of IL-6, a potent inflammatory cytokine inducing hepatic acute phase reactant production, has been associated with improvement of endothelial function, expressed as FMD, arterial stiffness and AIx, in open-label RCTs (40, 41). Interestingly, no changes in carotid IMT were reported (42), suggesting that rapid suppression of inflammation exerts more pronounced effect on endothelial function and that longer follow-up may be needed to detect significant changes of structural arterial wall damage. Conversely, B-cell blockade with rituximab was associated with improvement of carotid IMT in a pilot study without exerting significant effect on arterial stiffness and AIx in open label studies (43–45). Despite studies on atherosclerosis-prone mice demonstrated a favorable effect of abatacept in atherogenesis reduction, treatment with abatacept in humans was not associated with an improvement of surrogate measures of subclinical atherosclerosis, including aortic stiffness, AIx, carotid IMT, and plaques (40, 46).

## Do Anti-Rheumatic Therapies Lower the Risk of Cardiovascular Events?

### Conventional DMARDs

- Although no RCT explored the independent effect of MTX on major CV outcomes, robust evidence supports that patients treated with MTX are characterized by a significant lower risk of all CV events, myocardial infarction and stroke in comparison to RD patients not receiving MTX (12, 47, 48). The effect was more evident in responders to therapy and the pooled relative reduction resulted 28% for all CV events and 19% for myocardial infarction (12). Moreover, the evidence was stronger for overall reduction of CV morbidity and mortality and weaker for stroke risk reduction (12).
- As observed with MTX, no randomized studies evaluated the risk of CV diseases in RD patients treated with non-MTX non-biologic therapies. Observational data suggest that long exposure to leflunomide and sulfasalazine may be associated with a reduced risk of all CV events and myocardial infarction (38, 46).

### TNFα Inhibitors

- Meta-analysis of cohort studies demonstrated that use of TNFα-i in RA patients is associated with a 30% relative reduction in all CV events and a 41% reduction of myocardial infarction in comparison to other non-biologic therapies (12). Subsequent systematic literature review of different studies confirmed the safety of biologic therapies in RDs patients with respect to CV outcome (40). However, the high variability in study design, CV outcome definition, populations enrolled and disease activity hamper data interpretation and makes it difficult to compare results among studies (40).
- As observed for MTX, the favorable effect on CV outcome may depend on clinical response since a lower incidence of myocardial infarction has been observed in responders to therapy (49).
- A recent prospective study with a longer follow-up (median 5 years) demonstrated that TNFα-i therapy in RA patients is associated with a significant reduction of 39% in the risk of myocardial infarction in comparison to DMARD therapy (50). This is the first demonstration that duration of TNFα-i exposure may be associated with reduction of CV risk in these patients and suggests that stable suppression of inflammation and disease activity control are mandatory targets in the prevention of CV disease risk.
- A prospective analysis of the same cohort depicted that, compared to DMARDs, ever-exposure to TNFα-i therapy is not associated to a significant effect on the risk of first ischemic stroke over a median period of 5 years. Although not statistically significant, there was a trend toward a reduction in mortality at 30 days and at 1 year following the event among patients treated with TNFα-i at stroke occurrence compared to the other group (51). This may suggest different and still unexplored pathogenic mechanims underlying ischemic cerebrovascular events in RA patients.

### Other Non-TNFα-i Therapies

- Unfavorable lipid profile has been observed following TCZ therapy. However, pooled analysis of clinical trials and post-marketing safety data suggest that the CV disease risk in TCZ users is comparable to the risk associated with other biologic therapies (52–54). Indeed, a clear inverse relationship, known as the “lipid paradox,” has been demonstrated between lipid levels and CV risk in RA patients with an increased risk of CV disease also in patients with low total cholesterol and low-density lipoprotein (LDL) levels in the setting of active inflammation (55). Despite the global increase in LDL, total cholesterol, and triglyceride levels following the reduction of inflammatory burden, a favorable anti-inflammatory change of high-density lipoprotein composition and function has been demonstrated following tocilizumab administration, thus suggesting its positive net effect on CV risk (56).
- Abatacept may be associated with lower risk of myocardial infarction in comparison with TNFα-i. A retrospective study enrolling RA patients initiating biologic therapies, patients treated with abatacept were characterized by a lower risk of myocardial infarction in comparison to patients on TNFα-i therapy (57). Interestingly, these data have been recently confirmed in a large population-based cohort of RA patients demonstrating that abatacept was associated with a significant 29% reduced risk of a CV composite endopoint (myocardial infarction, stroke/transient ischemic attack, and coronary revascularization) when compared with TNFα-i therapy, in particular in patients with diabetes mellitus (58).
- Data on CV outcome in patients treated with rituximab are scarce. Observational studies did not observe significant differences in CV event rates in patients treated with rituximab in comparison to TNFα-i therapy or abatacept (40, 46).
- The period passed from the introduction of anti IL-12/23 targeted therapies is too short to draw hypothesis on their effect on CV outcome (40, 59).

## Open Questions and Future Directions

Despite broad evidence suggests that non-biologic and biologic therapies may be associated with a reduced risk of CV events and more favorable CV outcome in RD patients, several points should be considered in data interpretation, suggesting caution in their feasibility.

- The high variability in study designs and inclusion/exclusion criteria, in disease characteristics (grade of activity, seropositivity, duration, concomitant CV risk factors, concomitant therapies as non-steroidal anti-inflammatory drugs), in CV event definition, and in cohort enrolled represent a major limit to consider.
- The median follow-up of almost all studies was too short to effectively detect a significant reduction of long-term CV events. Similarly, the variable follow-up across studies makes it difficult to verify the durability of therapy effect on subclinical atherosclerosis measure improvement.
- The application in many studies of surrogate markers of atherosclerosis to estimate CV disease risk due to the low number of CV events, which limited statistical significance detection, remains an important limit in the interpretation of study results.
- Further studies are needed to investigate if the reduction of CV risk is a direct effect of these targeted therapies on atherosclerotic process or an indirect manifestation of the general reduction of systemic inflammation and disease activity.
- Research should focus on evaluation of drug-specific class effects on CV disease risk in order to enable better and personalized use of targeted therapies according to patient CV risk phenotype and disease characteristics.
- Further studies are needed to more deeply elucidate the contribution of inflammation to the pathophysiology of atherosclerosis in RDs and to identify specific non-invasive biomarkers to be employed as tool to identify patients with higher CV risk and guide therapy selection.
- The effect of targeted therapies on CV risk as well as pathogenic mechanisms leading to atherosclerotic damage in patients with SA and PsA should be further investigated.
- Larger, prospective studies with longer follow-up and RCTs with hard CV end-points are urgently needed to better characterize the CV outcome in these patients.
- Specific CV disease screening by validated CV risk score in RD patients should be implemented in order to quantify the CV long-term outcome and guide the better primary and secondary CV preventive therapeutic strategy.
- Despite advances in the treatment of these chronic RDs and better control of disease activity, CV-related mortality remains elevated in these patients. Under-recognition and suboptimally treatment of CV risk factors in association with the unavailability of validated treatment recommendations represent major causes for the lack of proper CV risk management in usual clinical care.

## Author Contributions

EB wrote the whole manuscript. RG revised and approved the final manuscript draft. All Authors revised and approved the manuscript.

### Conflict of Interest Statement

The authors declare that the research was conducted in the absence of any commercial or financial relationships that could be construed as a potential conflict of interest.
